# Prognostic Nutritional Index (PNI) and Neutrophil to Lymphocyte Ratio (NLR) as Predictors of Short-Term Survival in Patients with Advanced Malignant Biliary Obstruction Treated with Percutaneous Transhepatic Biliary Drainage

**DOI:** 10.3390/jcm11237055

**Published:** 2022-11-29

**Authors:** Milos Zakosek, Dusan Bulatovic, Vedrana Pavlovic, Aleksandar Filipovic, Aleksa Igic, Danijel Galun, Darko Jovanovic, Jelena Sisevic, Dragan Masulovic

**Affiliations:** 1 Center for Radiology, University Clinical Center of Serbia, 11000 Belgrade, Serbia; 2 Institute of Medical Statistics and Informatics, Faculty of Medicine, University of Belgrade, 11000 Belgrade, Serbia; 3 Faculty of Medicine, University of Belgrade, 11000 Belgrade, Serbia; 4 HPB Unit, Clinic for Digestive Surgery, University Clinical Center of Serbia, 11000 Belgrade, Serbia; 5 Clinic of Urology, University Clinical Center of Serbia, 11000 Belgrade, Serbia

**Keywords:** biliary obstruction, drainage, palliative, interventional radiology, oncology

## Abstract

Background: Effective biliary tree decompression plays a central role in the palliation of malignant biliary obstruction (MBO). When endoscopic drainage is unfeasible or unsuccessful, percutaneous transhepatic biliary drainage (PTBD) is the method of choice and preferred treatment approach in advanced hilar MBO. The prognostic nutritional index (PNI) reflects the patient’s immunonutritional status, while the neutrophil to lymphocyte ratio (NLR) reflects the patient’s inflammation status. The aim of the present study was to evaluate the prognostic value of preprocedural PNI and NLR on short-term survival in the advanced stage MBO population threatened with PTBD and to characterize the differences in immunonutritional and inflammatory status between 60-day survivors and non-survivors, as well as analyze other variables influencing short-term survival. Methods: This single-center retrospective study was conducted on patients undergoing palliative PTBD caused by MBO as a definitive therapeutic treatment between March 2020 and February 2022. After the procedure, patients were followed until the end of August 2022. Results: A total of 136 patients with malignant biliary obstruction were included in the study. Based on receiver operating characteristic (ROC) curve analysis, optimal cut off-values for NLR (3) and PNI (36.7) were determined. In univariate regression analysis, age, absolute neutrophil count, albumin level, NLR ≤ 3, and PNI ≥ 36.7 were significant predictors of 60-day survival. Level of obstruction and PNI ≥ 36.7 were statistically significant independent predictors of 60-day survival in a multivariate regression model. Using PNI ≥ 36.7 as a significant coefficient from the multivariate regression model with the addition of NLR ≤ 3 from univariate analysis, a 60-day survival score was developed. Conclusions: PNI and NLR are easy to calculate from routine blood analysis, which is regularly conducted for cancer patients. As such, they represent easily available, highly reproducible, and inexpensive tests capable of expressing the severity of systemic inflammatory responses in patients with cancer. Our study highlights that preprocedural PNI and NLR values provide predictors of short-term survival in patients with MBO treated with palliative PTBD. In addition, the proposed 60-day survival score can contribute to better selection of future candidates for PTBD and recognition of high-risk patients with expected poor outcomes.

## 1. Introduction

Malignant biliary obstruction (MBO) occurs when a tumor in or adjacent to bile ducts impairs bile flow from the liver to the intestinal tract [1]. It is often incurable and associated with very poor prognosis [2]. The most frequent causes of MBO include pancreatic adenocarcinoma, cholangiocarcinoma, gallbladder adenocarcinoma, liver metastasis, ampullary and duodenal adenocarcinoma, gastric cancer, hepatocellular carcinoma, and compressive metastatic hilar lymph nodes [3,4]. Jaundice is the main sign of bile duct obstruction. It develops in 70–90% of patients with important consequences, such as pruritus and weakening of cellular immunity, which allows tumor and metastasis progression, deterioration of coagulation due to decreased absorption of lipid-soluble vitamin K, and increased risk of infection [3,5,6]. In addition to jaundice, cholangitis and sepsis may also occur [5].

At the time of diagnosis, only a minority of patients with early-stage disease fit the criteria for curative surgery, while the rest, which are considered as nonsurgical candidates, are treated with chemoradiotherapy with the intent of downstaging the tumor or with strictly palliative procedures. Effective biliary tree decompression plays a central role in the palliation of MBO, with the goal of improving malabsorption and quality of life, preventing cholangitis, enabling patients to receive chemotherapy, and prolonging survival [7,8]. The first choice of treatment in palliation of MBO is endoscopic biliary drainage (EBD). Endoscopic placement of a biliary stent is a minimally invasive procedure that obviates the need for an exteriorized catheter and avoids complications like pain or catheter dislodgement [9]. However, due to duodenal obstruction or previous surgery that has changed the anatomy, EBD is not always feasible. In such cases, and when endoscopic drainage is unsuccessful, percutaneous transhepatic biliary drainage (PTBD) is the method of choice [2]. Furthermore, in patients with advanced unresectable hilar malignant biliary obstruction, a percutaneous approach is preferred and superior to endoscopic drainage [10].

It is increasingly recognized that the prognosis of patients with cancer is influenced not only by the oncological characteristics of the tumor, but also by the conditions of the host. The relationship between inflammatory and immunonutritional status and the prognosis of patients with cancer has attracted much attention in recent years. The prognostic nutritional index (PNI) is determined by combining values of the serum albumin level and absolute peripheral blood lymphocyte count. It reflects the nutritional and immunologic status of the patient. In recent years, several studies have shown its correlation with postoperative complications and cancer outcomes in different solid organ cancers, such as gastric cancer, non-small and small cell lung cancer, pancreatic cancer, colorectal cancer, hepatocellular carcinoma, esophageal cancer, and renal cell carcinoma [11,12,13,14,15,16,17,18,19]. The neutrophil to lymphocyte ratio (NLR), comprising the ratio between neutrophil and lymphocyte counts, is a simple and effective biomarker for observing the inflammatory state of the immune system [20]. Multiple studies published in the literature have shown the prognostic value of NLR in colorectal cancer, gastric cancer, lung cancer, and pancreatic cancer [21,22,23,24,25]. In addition to malignant diseases, PNI and NLR are extensively investigated in chronic conditions such as vascular and kidney diseases, where systemic inflammation plays a role in the progression of the disease and unfavorable outcome [26,27,28,29]. PNI reflects the patient’s immunonutritional status, while NLR reflects the patient’s inflammation status, both of which are easily available, highly reproducible, and inexpensive tests obtained via routine blood analysis [30].

To date, the prognostic value of PNI and NLR on survival is unclear in patients with advanced MBO in whom PTBD was performed, with a paucity of data reported in the current literature. Exploring the association between immunonutritional and inflammatory status, along with other clinical features, and survival in a population with advanced MBO who underwent PTBD is pivotal for recognizing individuals at highest risk for poor outcomes. The aim of the present study was to evaluate the prognostic value of preprocedural PNI and NLR on short-term survival in this population and characterize the differences in immunonutritional and inflammatory status between 60-day survivors and non-survivors, as well as analyze other variables influencing short-term survival.

## 2. Materials and Methods

### 2.1. Study Design

This single-center retrospective study was conducted on patients undergoing palliative PTBD caused by MBO as a definitive therapeutic treatment. All procedures were performed between March 2020 and February 2022 at the University Clinical Center of Serbia, Belgrade, Serbia. After the procedure, patients were followed until the end of August 2022. The inclusion criteria were: (a) patients with clinical, radiological, or histopathological diagnosis of MBO, (b) patients not eligible for curative surgery due to advanced disease, poor medical condition, or rejection to operate, (c) patients in whom EBD was not indicated or was unsuccessful, and (d) patients in whom PTBD was successfully performed. The exclusion criteria were: (a) patients who underwent postprocedural surgical treatment of biliary obstruction, and (b) patients lost to follow-up or incomplete medical data. Short-term survival was evaluated after the procedure, collocating the patients into two groups based on 60-day survival: “survivors” and “non-survivors”.

### 2.2. Data Collection

Data were retrospectively obtained from the hospital’s health informational system using patient medical records and archived images. Basic demographic, clinical, and PTBD procedural data were obtained for all patients: age, gender, cause of biliary obstruction, location of biliary obstruction (proximal—intrahepatic and hilar obstruction, or distal, subhilar obstruction), laboratory parameters (preprocedural absolute neutrophil and lymphocyte count, preprocedural serum albumin levels and serum bilirubin levels before and within 7 days after the procedure), insertion site of PTBD (right/left), presence of metastatic disease in liver, assessment of clinical success, complications associated with intervention, and obtaining of postprocedural chemotherapy or stereotactic body radiation therapy (SBRT) in addition to chemotherapy. Clinical success was defined as a decrease in serum bilirubin levels of more than 20% within 7 days after PTBD compared with preprocedural bilirubin levels. Survival was determined as the period between initial PTBD and the patient’s death. PNI was computed using the presented formula: serum albumin value (g/L) + 5 × absolute peripheral blood lymphocyte count (×10^9^/L). NLR was calculated as the neutrophil count divided by the lymphocyte count. For classifying and grading the complications, we used the CIRSE Classification System for Complications [31].

### 2.3. Study Outcomes

The primary endpoint of the study was to evaluate the prognostic value of preprocedural PNI and NLR on 60-day survival in this population. A second aim was to analyze other variables influencing short-term survival. Additionally, a 60-day survival score was developed using cut-off values for PNI and NLR obtained from the receiver operating characteristic (ROC) curve analysis.

### 2.4. Ethical Approval

The protocol of the study was approved by the institutional review board, namely the Ethics Committee of the University Clinical Center of Serbia.

### 2.5. Procedure

All procedures were carried out by an experienced team of interventional radiologists in a referral center for interventional radiology procedures of the hepatobiliary tract in Serbia. Written informed consent was obtained from all patients before the procedure. We defined PTBD as technically successful if either internal-external drainage or external drainage only was accomplished.

All patients had a preprocedural CT or MR examination with the aim of determining the cause and level of obstruction, and to choose whether to perform PTBD via the right or left liver lobe access site. The procedures were performed under conscious sedation. Initial puncture of the biliary tract was performed under ultrasound guidance (US) and fluoroscopic guidance was used for the rest of the procedure. The most suitable peripheral bile duct for puncture was determined based on preprocedural US and cross-sectional imaging in order to avoid transgressing the intervening vital structures (stomach, colon), vascular elements, and metastasis, if present. Following local anesthetic injection (10 mL of xylocaine/carbocaine), a small skin incision was made at the access site. The percutaneous access set, consisting of a 22-gauge Chiba needle, 0.018” nitinol guidewire, and 4F introducer sheath (Neff Percutaneous Access Set, Cook Incorporated, Bloomington, IN, USA) or 19-gauge introducer sheath/needle (Argon Medical Systems Inc., Athens, TX, USA), was used to puncture the bile duct. Puncture was performed under US guidance (Xario 200, Canon Medical Systems Corporation, Otawara, Japan) using in-plane free-hand technique. The right liver lobe was punctured using intercostal access, whereas the left liver lobe was punctured via subxiphoid epigastric access. After needle insertion, fluoroscopy was used to confirm the location of the needle by removing the inner needle and injecting a small amount of contrast medium through the outer sheath. Effort was made to minimize instrumentation and limit contrast opacification of the bile ducts in the undrained biliary system in order to reduce the infectious complication rate. After opacification of the biliary system, 0.035” hydrophilic guidewire (Radiofocus Guide Wire M, Terumo Corporation, Tokyo, Japan) was introduced in the intrahepatic duct and a series of maneuvers were made to cross the biliary stricture and place the guidewire into the duodenum or the efferent jejunal loop, in case of bilio-enteric anastomoses, with the intention of placing the internal biliary drainage (Biliary Drainage Catheter, Cook Incorporated, Bloomington, IN, USA). If attempts to cross the stricture were unfeasible, the hydrophilic guidewire was swapped for 0.035” stiff wire (Inqwire, Merit Medical, Galway, Ireland) and after dilatation of the transhepatic tract (Dilator, Cook Incorporated, Bloomington, IN, USA), an external drainage catheter (Dawson-Mueller Multipurpose Drainage Catheter, Cook Incorporated, Bloomington, IN, USA) was placed above the level of obstruction. A small amount of contrast medium was injected and fluoroscopy was used to confirm the correct placement and function of the drainage catheter. To minimize the rate of infectious complications, antibiotic prophylaxis covering the upper gastrointestinal flora was used. After successfully performance of PTBD, patients were followed for occurrence of complications at regular intervals in control examinations. Furthermore, patients were referred to the multidisciplinary tumor board to decide if they were eligible for chemotherapy or SBRT in addition to chemotherapy.

### 2.6. Data Analysis

The descriptive statistics, including means, medians, standard deviations, and percentiles for numerical variables and numbers and percentages for categorical variables, were used to characterize the study samples. Associations between categorical data were evaluated using the Pearson chi-square test. Student’s *t*-test or the Mann-Whitney U test were used for numerical data to evaluate differences between survivors and non-survivors after 60 days. Univariate and multivariate logistic regression analyses were used to establish factors related to overall mortality. Significant variables from univariate analysis were included in multivariate regression, with overall mortality after 60 days as the outcome. The results were expressed as relative risk and corresponding 95% confidence interval (CI). Model discrimination performance was tested by means of sensitivity, specificity, and positive and negative predictive values. C statistic, representing the area under the receiver operating characteristic (ROC) curve, was used for overall assessment of the predictive model. In all analyses, the level of statistical significance was set at *p* ≤ 0.05. SPSS version 25 statistical software (Chicago, IL, USA) was used to perform the statistical analysis.

## 3. Results

### 3.1. Patients

A total of 136 patients with malignant biliary obstruction were included in the study. More than half of the study population was male (52.9%) and the average age was 65.7 ± 11.7 years. The most frequent tumor entity causing MBO was pancreatic head cancer, present in 27.2% of patients, while Klatskin tumors were present in 21.3% of patients, and 18.4% of patients had liver hilum metastasis. More than one third of patients had distal obstruction (38.2%) and liver metastasis (39.0%). The overall median survival in our study was 62 days (1–434), and overall median follow-up for survivors was 389 days (196–590).

Patients who died were older (*p* = 0.047), had higher bilirubin levels within 7 days after PTBD (*p* = 0.010), higher values of absolute neutrophils (*p* = 0.003), lower albumin levels (*p* = 0.001), and did not receive further treatment (*p* = 0.001). In addition, patients who died had lower PNI (*p* = 0.004) and higher NLR (*p* = 0.021) scores. The detailed demographic and clinical characteristics of the study population overall and according to the survival groups are presented in Table 1.

Among 136 patients, 13 (9.6%) patients had intra/periprocedural complications or complications in the follow-up period. The most frequent complication was catheter dislodgement in 6 patients (4.4%). In such cases, during the same session, the catheter was repositioned in the original position or the catheter was exchanged (grade 1). Minor haemobilia, a grade 1 complication, was present in 3 patients (2.2%), which was solved within the same session and no additional therapy or postprocedural sequelae were required. Cholangitis and fever were present in 3 patients (2.2%), a grade 3 complication, which required additional postprocedural therapy or prolonged hospital stay (≥48 h) without postprocedural sequelae. Bleeding requiring surgical intervention was present in 1 case (0.7%), a grade 4 complication. There were no complications causing death.

In our study, 49 patients (36%) received further treatment, 45 patients (33.1%) received chemotherapy, and 4 patients (2.9%) received SBRT in addition to chemotherapy.

### 3.2. Statistical Analysis

#### 3.2.1. Cut Off-Values for NLR and PNI Scores

**Figure 1:**
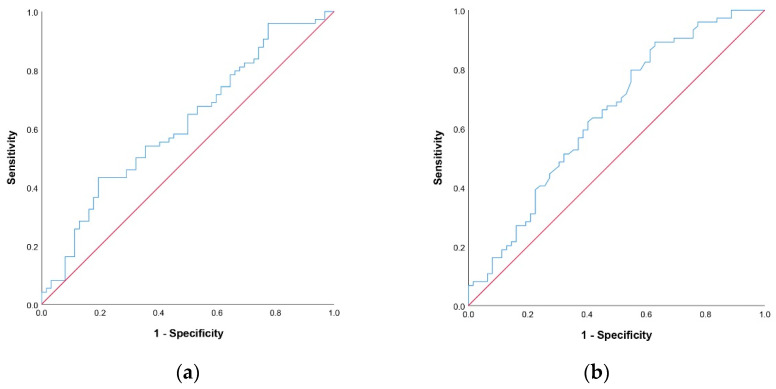
ROC curve for NLR (**a**) and PNI (**b**).

Based on the ROC curve analysis (Figure 1), optimal cut off-values for NLR and PNI scores were determined. The area under the curve (AUC) value for NLR was 0.615 and the AUC value for PNI was 0.643 (*p* = 0.021 and *p* = 0.004, respectively). Diagnostic performance of NLR score used to predict survival was tested and the sensitivity, PPV, specificity, and NPV of NLR ≤ 3 were: 28.4%, 67.7%, 83.9%, and 49.5%, respectively. The sensitivity, PPV, specificity, and NPV of PNI ≥ 36.7 were: 66.2%, 62.8%, 53.2%, and 56.9%, respectively.

#### 3.2.2. Univariate and Multivariate Logistic Regression Analysis

The results of univariate and multivariate logistic regression analyses with 60-day survival as the dependent variable are presented in Table 2. In univariate regression analysis, age (*p* = 0.049), absolute neutrophil count (*p* = 0.026), albumin level (*p* = 0.002), NLR ≤ 3 (*p* = 0.056), and PNI ≥ 36.7 (*p* = 0.023) were significant predictors of 60-day survival. Level of obstruction (*p* = 0.027) and PNI ≥ 36.7 (*p* = 0.011) were statistically significant independent predictors of 60-day survival in the multivariate regression model.

### 3.3. 60-Day Survival Score

**Figure 2:**
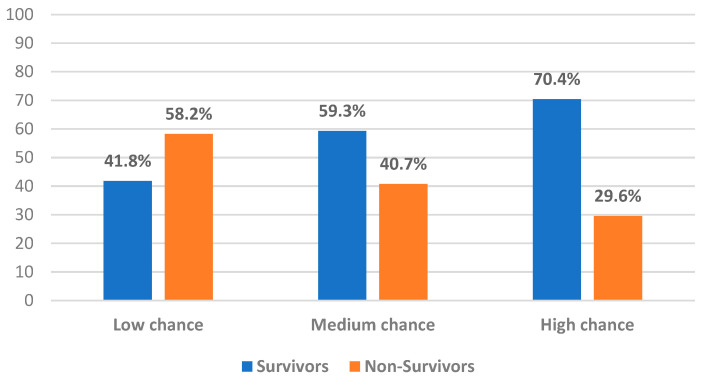
Distribution of 60-day survival score in the study population.

Using PNI ≥ 36.7 as the significant coefficient from the multivariate regression model with the addition of NLR ≤ 3 from the univariate analysis, the chance prediction score was developed, as seen in Table 3. The newly developed 60-day survival score was determined by assigning 0 points if NLR > 3 and PNI < 36.7, 1 point was assigned if NLR ≤ 3 and PNI < 36.7 or NLR > 3 and PNI ≥ 36.7, and 2 points were assigned if NLR ≤ 3 and PNI ≥ 36.7. Finally, based on the survival chance prediction score, the population was divided into chance categories for survival: low chance (score 0) and medium chance (score 1). In addition, patients were considered at high chance of survival with a risk score of 2. The distribution of 60-day survival scores in our study population is shown in Figure 2.

## 4. Discussion

The objective of this study was to investigate the prognostic value of PNI and NLR on short-term survival in patients undergoing palliative PTBD for advanced MBO. The results of our study in a retrospective cohort consisting of 136 patients showed that both PNI and NLR were significant predictors of 60-day survival. Based on the ROC curve analysis, the optimal cut off-values for NLR and PNI scores were 3 and 36.7, respectively. The short-term survival of patients with PNI ≥ 36.7 was observed to be significantly higher than that of patients with PNI < 36.7. Also, patients with NLR ≤ 3 had significantly higher survival than patients with NLR > 3. This study also demonstrated that PNI is superior to NLR as a predictor of survival.

PNI, calculated based on total lymphocyte counts and serum albumin levels, reflects the nutritional and immunological status of cancer patients. Lymphocytes are an essential part of cell-mediated antitumor immune reactions and tumor immunological surveillance by means of inducing cancer cell apoptosis and inhibiting cancer cell proliferation [32,33]. Low lymphocyte counts result in poor immunological responses in the tumor microenvironment and cancer progression. At the same time, malnutrition occurs very frequently in cancer patients, with almost 40 to 80% of all cancer patients being malnourished during the course of the disease [34]. Serum albumin level in PNI is an indicator of nutritional status. Low albumin levels are associated with malnutrition and weight loss, which has a negative impact on the survival and recovery of cancer patients. Consequently, as a result of the reasons stated above, a low PNI value will correlate with unfavorable survival in cancer patients.

Due to heterogeneity among patients with MBO and diverse causes of obstruction, cut-off values for PNI and NLR vary among different studies. A meta-analysis involving 11 studies of patients with pancreatic cancer indicated that lower PNI values were significantly correlated with poorer overall survival [35]. Cui et al. reported that PNI was an independent predictor of survival in patients with advanced hilar cholangiocarcinoma treated with percutaneous transhepatic biliary stenting in combination with ^125^I seed intracavitary irradiation [36]. In the study by Salati et al. assessing the impact of PNI on survival and treatment response in advanced biliary cancer receiving chemotherapy, the same cut-off value for PNI was established as in our study using ROC analysis [37]. Based on the multivariate analysis in our study, PNI was found to be an independent prognostic factor and lower values of PNI correlated with worse short-term survival, findings that were consistent with previously mentioned studies. In terms of nonmalignant conditions, a study by Kaller et al. reported that PNI was an independent predictor of adverse outcomes in patients with arteriovenous fistula maturation failure, with lower PNI values associated with all adverse events, findings that aligned with those of our study. ROC curve analysis was utilized to determine the optimal cut-off value for PNI, which differed according to the examined adverse event, and a value of 38.65 for early thrombosis was closest to the PNI value found in our study [26].

NLR is calculated based on neutrophil and lymphocyte counts and serves as an effective biomarker of the inflammatory state of the immune system [20]. Neutrophils secret cytokines and chemokines, which inhibit cytotoxic immune cells and thus suppress the host immune response to cancer [38]. They also promote cancer growth and metastasis via angiogenic action and growth factors [39]. On the other hand, lymphocytes play an important role in antitumor immune reactions and immune surveillance through the induction of cancer cell apoptosis and inhibition of proliferation and migration of tumor cells [32,33]. NLR mirrors an equity between the protumor and antitumor inflammatory status of patients with cancer, whereas any change in the neutrophil to lymphocyte ratio can be associated with tumor progression [40,41].

In a systematic review and meta-analysis conducted by Liu et al., the prognostic value of NLR in cholangiocellular carcinoma was investigated in 32 studies. Pooled outcomes of these studies showed that high NLR prior to treatment was a prognostic factor for both poor overall survival and disease-free survival with meaningful hazard ratio values. The subgroup analysis indicated that this association was not substantially affected by treatment modality, NLR cut-off value, age, nor sample size of the included studies [42]. Among 32 investigated studies, 9 studies used the same cut-off value for NLR as we defined in our study [42,43]. A study by Iwai et al. revealed that NLR, with a cut-off value of 3.74, was an independent prognostic factor in patients with unresectable pancreatic cancer, including those without indication for chemotherapy. High NLR values were independently associated with worse overall survival [44]. In our study, a NLR cut-off value of 3 was consistent with the level range in previous studies. Even though univariate analysis in our study proved NLR to be a significant predictor of short-term survival, the multivariate regression analysis suggested that NLR is not an independent prognostic factor, thus asserting the clinical significance of combining preprocedural values of NLR and PNI in our proposed 60-day survival score for discerning patients with expected poor outcomes. High NLR values have been associated with unfavorable outcomes in patients with carotid artery and end-stage kidney disease [27,28]. According to the results reported by Niculescu et al., a high preoperative value of NLR was a strong predictor of 12-month restenosis and mortality following carotid endarterectomy. In this study, the optimal NLR cut-off value for restenosis was 3.47, a finding that aligned with our results [28]. Another study examined the predictive value of inflammatory markers on outcomes of patients with end-stage kidney disease, demonstrating that high NLR values were associated with 30-day mortality, number of days in hospital, and number of dialysis sessions per patient [27].

Most patients after biliary drainage cannot proceed to further treatment, which reflects their poor survival and the disease’s unavoidable course [45]. In our study, the median survival period after PTBD was 62 days, with an upper limit of 434 days. Reported post-PTBD survival varies from 44 days to 185 days and our median survival time was in the middle range of the literature data [46,47]. In the study by Iwasaki et al., the reported mean survival of patients with biliary obstruction caused by metastases from nonbiliary and nonpancreatic cancers was similar to that in our study [48]. In the study conducted by Niemela et al., a total of 643 patients with malignant biliary obstruction were treated with percutaneous drainage in a tertiary-level university hospital and the median overall survival was 2.6 months, which was similar to that in our study [49]. Poor expected survival of patients with MBO was the reason why we focused on short-term 60-day survival in our study.

Clinical success rates of PTBD vary between 75 and 98% in relevant studies [2]. Zhang et al. had clinical success in 76.5% of cases using the same threshold of more than 20% decrease in serum bilirubin levels within 7 days after PTBD [47]. In our population, out of 136 patients, 114 patients had clinical success, or 83.8%. This result is in accordance with the previous literature. Percutaneous transhepatic biliary drainage complication rates vary in the literature between 4% and 12% [4]. The complication rate in our study was not a statistically significant predictor of 60-day survival, was in the range stated in previous literature, and aligned with published standards of practice [4,50].

Previous studies have investigated other preprocedural prognostic factors that can predict survival in patients treated with PTBD. PTBD is a lifesaving but technically challenging procedure that can also lead to significant iatrogenic harm [51]. By determining predictive factors for poor survival through analysis of pre-PTBD clinical, radiological, and laboratory data, we can identify patients in whom PTBD will not improve wellbeing and survival, ultimately avoiding an invasive procedure in such a scenario. In our study, univariate analysis showed that age is a predictor of short-term survival, which was consistent with the findings of Rees et al. [52]. In previous studies, the serum albumin value was established as one of the most important factors in predicting short-term survival in patients undergoing PTBD, which was confirmed in our study [46,53]. In the study by Zhang et al. conducted on 102 patients, serum bilirubin level following PTBD was associated with survival, a finding that aligned with our study [47]. In our population, multivariate regression analysis indicated that level of obstruction was a significant predictor of 60-day survival, which was contrary to the findings of Zhang et al. [47]. The most frequent tumor entity with distal MBO in our population was advanced pancreatic cancer, which has poor prognosis. We are of the opinion that this may have contributed to our result. In the study conducted by Kasuga et al., 6 prognostic factors were independently associated with poor prognosis: poor performance situation, presence of multiple liver metastases, presence of ascites, receiving multiple chemotherapy before intervention, undifferentiated tumor type, and high level of serum carcinoembryonic antigen 19-9 [54]. However, the presence of liver metastasis did not demonstrate statistical significance in our study, which was contrary to other literature data [46,54]. As the focus of our study was on short-term 60-day survival, in our opinion, this may be the underlying cause of conflicting results with other literature data, since the presence of liver metastasis would most likely be of significance for long-term overall survival.

Many studies in the literature pointed out that PTBD can lead to significant bilirubin reduction and enable patients to receive chemotherapy, which prolongs survival after the procedure [15,47,54]. In our study, 33.1% received chemotherapy and 2.9% received SBRT in addition to chemotherapy, which was within the range of previous studies [9,46,55]. Total serum bilirubin levels before the procedure were rather high, with a median value of 351.0 μmol/l (287.5–466.0), and the median total serum bilirubin level before the procedure was even higher in the non-survivor group at 366.7 μmol/l. As demonstrated by Levy et al., patients with high preprocedural bilirubin levels achieved clinically relevant bilirubin reduction but had shorter survival [56]. With such high values of baseline total serum bilirubin levels in our study, patients with advanced-stage disease and liver impairment were expected to have poor survival and less likely to be regarded as candidates for chemotherapy. Moreover, receiving chemotherapy for uncurable disease also depends on age, performance status, co-morbidities, and personal decision.

Based on PNI and NLR cut-off values and univariate and multivariate logistic regression analyses, a 60-day survival score was proposed and developed with the aim of obtaining an early warning for possible poor outcomes of the procedure. The score enabled stratification of candidates for PTBD into low, medium, and high chances for survival. Other predictive models have been created and reported in the literature to predict early mortality in patients treated with PTBD, which include multiple demographic, preprocedural, blood biochemical (NLR among others), and tumor-related parameters in the analysis. Despite their high accuracy, these models are complicated to use and require further optimization [57]. We believe that our proposed score can aid clinicians in recognizing candidates who will benefit most from the procedure. However, a multi-center prospective study is needed to confirm and validate our results on a larger number of patients. For that reason, we advise to not rely solely on the proposed score in the clinical decision-making process, but to use it in addition to other determined predictive survival factors described in the literature.

Our study was limited due to its retrospective nature, small sample size, and inclusion of patients with different primary tumors. Nevertheless, the study included patients treated in a consistent fashion in a single tertiary referral center over a period of 2 years. A multi-center prospective study is needed to confirm our results on a larger number of patients and assess a unified ideal cut-off value using subgroup analysis by tumor entity.

## 5. Conclusions

Patients in whom palliative PTBD is performed have poor prognosis, with an expected survival of only a few months after drain insertion. Our study highlights that preprocedural PNI and NLR values are predictors of short-term survival in patients with MBO treated with palliative PTBD. PNI and NLR are easy to calculate from routine blood analysis, which is regularly conducted for cancer patients. As such, they represent easily available, highly reproducible, and inexpensive tests capable of expressing the severity of systemic inflammatory responses in patients with cancer. To the best of our knowledge, this is the first study to use both NLR and PNI as prognostic factors in patients with advanced malignant biliary obstruction treated with palliative PTBD. Moreover, combining their preprocedural values with our proposed cut-off values, we developed a 60-day survival score. In our view, the proposed score can contribute to better selection of future candidates for PTBD and recognition of high-risk patients with expected poor outcome.

## Figures and Tables

**Table 1:** Demographic and clinical characteristics of study population, overall and according to the survival groups.

Variables	Total	60-Day Survival		
		Non-Survivors	Survivors	*p*
	n = 136	n = 62	n = 74	
Gender, male, n (%)	72 (52.9)	35 (56.5)	37 (50.0)	0.453
Age, mean ± sd	65.7 ± 11.7	67.9 ± 11.4	63.9 ± 11.7	0.047
Diagnosis, n (%)				
Klatskin tumor	29 (21.3)	11 (17.7)	18 (24.3)	
Periampullary tumor	13 (9.6)	5 (8.1)	8 (10.8)	
Liver hilum metastasis	25 (18.4)	10 (16.1)	15 (20.3)	
Pancreatic cancer	37 (27.2)	21 (33.9)	16 (21.6)	0.685
Gallbladder carcinoma	11 (8.1)	6 (9.7)	5 (6.8)	
Primary liver tumor (HCC, CCC)	11 (8.1)	4 (6.5)	7 (9.5)	
Gastric adenocarcinoma	10 (7.4)	5 (8.1)	5 (6.8)	
Level of obstruction, n (%)				
Proximal	84 (61.8)	33 (53.2)	51 (68.9)	0.061
Distal	52 (38.2)	29 (46.8)	23 (31.1)	
PTBD access site, n (%),				0.085
Right lobe	100 (73.5)	50 (80.6)	50 (67.6)	
Left lobe	36 (26.5)	12 (19.4)	24 (32.4)	
Complications, n (%)	13 (9.6)	6 (9.7)	7 (9.5)	0.966
Liver metastasis, n (%)	53 (39.0)	25 (40.3)	28 (37.8)	0.767
Further treatment, n (%)	49 (36)	1 (1.6)	48 (64.9)	0.001
Chemotherapy	45 (33.1)	1 (1.6)	44 (59.5)	
Chemotherapy and SBRT	4 (2.9)		4 (5.4)	
Preprocedural total bilirubin median (25th–75th percentile)	351.0 (287.5–466.0)	366.7 (297.9–462.0)	345.0 (286.9–474.4)	0.795
Postprocedural total bilirubin median (25th–75th percentile)	194.1 (81.4–282.7)	219.2 (116.8–317.4)	146.0 (68.0–244.8)	0.010
Absolute neutrophil count (10^9^/L), median (25th–75th percentile)	5.8 (4.3–7.9)	6.4 (5.0–8.9)	5.2 (3.8–7.0)	0.003
Absolute lymphocyte count(10^9^/L), median (25th–75th percentile)	1.2 (0.9–1.5)	1.2 (0.8–1.6)	1.2 (0.9–1.5)	0.993
Serum albumin, mean ± sd	32.3 ± 5.6	30.6 ± 5.8	33.7 ± 5.0	0.001
PNI, median (25th–75th percentile)	38.3 (34.0–43.5)	36.2 (32.0–41.6)	40.2 (35.5–45.5)	0.004
NLR, median (25th–75th percentile)	4.7 (3.2–7.8)	5.3 (3.6–10.1)	4.0 (2.9–6.6)	0.021

**Table 2:** Univariate and multivariate logistic regression analysis with 60 days survival as dependent variable.

Variable	OR	95% CI for OR	*p*
Univariate			
Gender	1.296	0.658–2.554	0.453
Age	0.970	0.941–1.000	0.049
Liver metastasis	0.858	0.426–1.726	0.767
Level of obstruction	0.513	0.255–1.034	0.062
PTBD access site	2.000	0.902–4.434	0.088
Preprocedural total bilirubin	1.000	0.998–1.002	0.955
Postprocedural total bilirubin	0.998	0.996–1.000	0.087
Absolute neutrophil count	0.895	0.812–0.987	0.026
Absolute lymphocyte count	1.225	0.839–1.790	0.293
Serum albumin level	1.116	1.042–1.195	0.002
NLR ≤ 3	0.429	0.180–1.022	0.056
PNI ≥ 36.7	2.230	1.115–4.462	0.023
Multivariate			
Level of obstruction	0.437	0.209–0.911	0.027
PNI ≥ 36.7	2.562	1.243–5.279	0.011

**Table 3:** 60-day survival score using cut-off values for NLR and PNI.

Values	Point	Event	60-Day Survival Score	Survival Chance
NLR > 3	0	NLR > 3 and PNI < 36.7	0	Low chance
NLR ≤ 3	1	NLR > 3 and PNI ≥ 36.7	1	Medium chance
PNI < 36.7	0	NLR ≤ 3 and PNI < 36.7	1	Medium chance
PNI ≥ 36.7	1	NLR ≤ 3 and PNI ≥ 36.7	2	High chance

## Data Availability

Anonymized study data are available from the corresponding author upon reasonable request.
